# Sex-related differences in human plasma NAD^+^/NADH levels depend on age

**DOI:** 10.1042/BSR20200340

**Published:** 2021-01-14

**Authors:** Luisa Schwarzmann, Rainer Ullrich Pliquett, Andreas Simm, Babett Bartling

**Affiliations:** 1 Department of Cardiac Surgery, Mid-German Heart Center, University Hospital Halle (Saale), Martin Luther University Halle-Wittenberg, Halle (Saale), Germany; 2 Department of Internal Medicine II, Medical Faculty, Martin Luther University Halle-Wittenberg, Halle (Saale), Germany; 3 Department of Nephrology and Diabetology, Carl Thiem Hospital, Cottbus, Germany; 4 Animal Health Management, Institute of Agricultural and Nutritional Sciences, Martin Luther University Halle-Wittenberg, Halle (Saale), Germany

**Keywords:** biological age, blood, NAD/NADH redox stage, sex

## Abstract

Nicotinamide adenine dinucleotide (NAD) is a coenzyme in metabolic reactions and cosubstrate in signaling pathways of cells. While the intracellular function of NAD is well described, much less is known about its importance as an extracellular molecule. Moreover, there is only little information about the concentration of extracellular NAD and the ratio between its oxidized (NAD^+^) and reduced (NADH) form in humans. Therefore, our study aimed at the analysis of total NAD and NAD^+^/NADH ratio in human plasma depending on sex and age. First, an enzymatic assay was established for detecting NAD^+^ and NADH in human plasma samples. Then, plasma NAD was analyzed in 205 probands without severe diseases (91 men, 114 women) being 18–83 years old. The total plasma NAD concentration was determined with median 1.34 µM (0.44–2.88 µM) without difference between men and women. Although the amounts of NAD^+^ and NADH were nearly balanced, women had higher plasma NAD^+^/NADH ratios than men (median 1.33 vs. 1.09, *P*<0.001). The sex-related difference in the plasma NAD^+^/NADH ratio reduces with increasing age, an effect that was more obvious for two parameters of the biological age (skin autofluorescence, brachial-femoral pulse wave velocity (PWV)) than for the chronological age. However, plasma values for total NAD and NAD^+^/NADH ratio did not generally alter with increasing age. In conclusion, human plasma contains low micromolar concentrations of total NAD with higher NAD^+^/NADH redox ratios in adult but not older women compared with same-aged men.

## Introduction

The coenzyme nicotinamide adenine dinucleotide (NAD) converts between its oxidized form, NAD^+^, and reduced form, NADH, thereby mediating the hydrogen transfer in metabolic reactions [4,40]. NADH is mainly generated by catabolic reactions, whereas NAD^+^ is generated by the mitochondrial electron transfer chain [4,40]. Therefore, the redox ratio of NAD^+^/NADH reflects the metabolic stage of cells [4,40]. In humans, NAD^+^ can be produced by three pathways: the kynurenine pathway synthesizing NAD^+^ *de novo* from the amino acid l-tryptophan, the Preiss–Handler pathway synthesizing NAD^+^ from nicotinic acid (niacin) or nicotinic acid riboside, and the salvage pathway synthesizing NAD^+^ from nicotinamide, an NAD metabolite [4,40]. As NAD^+^ precursors can be taken up from the extracellular space, the bioavailability of cellular NAD^+^ is improved by dietary supplementation with NAD^+^ precursors [10,33]. Moreover, physical exercise and caloric restriction change the cellular redox ratio of NAD^+^/NADH toward NAD^+^ [10].

The hydrogen transfer is not the sole function of NAD^+^. NAD^+^ also serves as cosubstrate of three classes of enzymes: poly-ADP-ribose polymerases like poly-ADP-ribose polymerase 1 (PARP-1), type III protein lysine deacylases (sirtuins) like Sirt1 and cyclic ADP-ribose synthases like cluster of differentiation (CD)38 [4,33]. These enzymes are NAD^+^ consumers because they cleave NAD^+^ at the glycoside bond between nicotinamide and ADP-ribose [4,33]. The salvage pathway then generates fresh NAD^+^ from nicotinamide [4,40]. Therefore, cellular NAD^+^/NADH ratios do not only reflect the metabolic cell stage but also the balance between NAD^+^ consumption and generation.

Among the NAD^+^ consumers, CD38 does not depend on intra- but on extracellular NAD^+^ because CD38 is an ectoenzyme located at the outer cell membrane [26]. In addition to lytic processes, NAD^+^ is actively released from cells via connexin-43 (Cx43) hemichannels [6,38]. Although nearly all cell types show an expression of Cx43, it is most expressed in cardiomyocytes and nerve cells [27], whereas CD38 is mainly expressed in lymphoid tissues [26]. As the reaction products of CD38, nicotinamide and ADP-ribose, are essential for the regulation of intracellular Ca^2+^, extracellular NAD^+^ plays an indirect role in the Ca^2+^ metabolism [26]. Moreover, the NAD^+^-dependent generation of ADP-ribose contributes to the ADP-ribosylation of cell surface proteins including the purinergic receptors P2X7 [24]. Other NAD^+^-cleaving ectoenzymes generating extracellular ADP-ribose could be CD157 belonging to the same gene family as CD38 [26] and the nucleotide pyrophosphatase/phosphodiesterase 1 [25].

Extracellular NAD^+^ is also believed to act directly via binding to purinergic receptors of the P1, P2X and P2Y families [15,23,30,31]. While P2X receptors cause an influx in Ca^2+^ and Na^+^ via activation of ion channels, P1 and P2Y receptors mediate G protein-mediated cell signaling [34]. Like CD38, purinergic receptors are mainly expressed in cells of the immune system [34] that again indicates the potential importance of extracellular NAD^+^ in the immune modulation. In addition, extracellular NAD^+^ could also modulate the vasotonus via the P1 purinergic receptors A2A as shown for isolated vessels [2]. The general importance of purinergic signaling in the cardiovascular system has been demonstrated in many studies [7].

In the last decade, the ‘old’ molecule NAD^+^ became of interest again because NAD^+^-consuming sirtuins could contribute to longevity through their beneficial effects on oxidative stress and inflammation [14]. As intra-cellular NAD^+^ is decreased in tissues of old animals [33] and humans [22,28,41,42], the NAD^+^-sirtuin axis eventually plays a significant role in the process of aging [18,19]. The lower activity of the salvage pathway might be reason of the lower NAD^+^ amounts in old tissues [13]. Therefore, dietary NAD^+^ precursors are subject of many clinical trials [10,33]. In contrast with tissue NAD, nearly nothing is known about the effect of aging and other parameters including sex on the extracellular NAD amounts and the NAD redox ratios in body fluids. Also, the plasma or serum concentrations described vary extremely (0.05–500 µM) [5,16,17,36,37].

The chronological age does not represent the biological age of humans because everyone ages at a different rate. Therefore, non-invasive measurements detecting at least some markers of the biological age became attractive in population studies. Among them is the non-invasive detection of the pulse wave velocity (PWV) assessing the age-associated stiffness of arterial vessels [3]. This is of special importance because most elderly people die worldwide of cardiovascular diseases [35]. Another non-invasive method is the detection of skin autofluorescence. The age-related increase in skin autofluorescence reflects the increasing accumulation advanced glycation end-products in the cutaneous extracellular matrix and, therefore, the skin aging [12].

An increase in the chronological or biological age could change the blood level of NAD and NAD^+^/NADH redox ratio in men and women. Therefore, our study analyzed the NAD status in plasma samples derived from a large normal human population depending on sex and age.

## Materials and methods

### Study population

A total of 205 probands between 18 and 83 years were included in our study which was approved by the Ethics Committee of the Medical Faculty, Halle (Saale). We reached a balanced distribution of the probands regarding sex and chronological age (Table 1). Women being pregnant or persons suffering from acute or severe chronic diseases were excluded from the study. These diseases were alcoholism, cancer, chronic inflammatory diseases, cold, diabetes mellitus type 1, fever, heart failure (New York Heart Association (NYHA) stage ≥ 2), liver cirrhosis of any stage, renal failure (Kidney Disease: Improving Global Outcomes (KDIGO) stage ≥ G3), and neurological disorders including dementia. Persons with common age-related diseases (i.e. hypertension, diabetes mellitus type 2) or other common diseases (i.e. hypothyroidism) were not excluded, unless they were in an uncontrolled condition. Although diabetes cannot be excluded as plasma NAD-modifying disease, only one person suffered from diabetes mellitus type 2. All participants gave their written informed consent for inclusion in the study, all study-related procedures and data analysis.

**Table 1:** General parameters of the study population depending on sex and chronological age

		Men (*n*=91)			Women (*n*=114)		
		Mean ± SD	Age dependency^1^		Mean ± SD	Age dependency^1^	
			+/–	R^2^		+/–	R^2^
**Chronological age**	Years	46.7 ± 17.5			48.2 ± 17.2		
**Biological age**							
Skin autofluorescence	aU	2.05 ± 0.48	+	0.598^###^	2.01 ± 0.48	+	0.422^###^
Brachial-femoral PWV	m/s	12.7 ± 3.92	+	0.556^###^	13.6 ± 5.20	+	0.605^###^
**Body mass index**	kg/m^2^	25.7 ± 3.37	+	0.051^#^	25.1 ± 3.37	+	0.071^#^
**Physical activity**^2^	score	1.87 ± 0.69		0.033	1.80 ± 0.76	–	0.085^##^
**Hand force**	kg	45.3 ± 9.94	–	0.173^###^	27.2 ± 5.37^***^	–	0.213^###^
**Pulse pressure**	mmHg	49.2 ± 11.9	+	0.228^###^	43.0 ± 12.7^***^	+	0.476^###^
**Medical drugs**	N	1.00 ± 1.58	+	0.344^###^	1.59 ± 1.90^*^	+	0.228^#^
**Plasma lysozyme**^3^	ng/ml	135 ± 41.9	+	0.091^#^	121 ± 32.5^*^	+	0.050^#^

*P<0.05 and ***P<0.001 vs. men as calculated by the Rank sum test or Student’s *t* test.

^#^*P*<0.05, ^##^*P*<0.01 and ^###^*P*<0.001 for coefficient R^2^ of the quadratic polynomial regression analysis.

1 Direct (+) or inverse (–) correlation with increasing age.

2 Self-assessment (1-low to 4-high).

3 Normal plasma levels < 1000 ng/ml [1].

Basal data were collected by use of a standardized questionnaire. The Vicorder® device (SMT medical GmbH, Würzburg, Germany) measured the PWV between brachial and femoral artery. The AGE Reader mu® (Diagnoptics Technologies, Groningen, Netherlands) detected the skin autofluorescence inside the forearm. Systolic and diastolic blood pressure were measured with an upper arm blood pressure monitor (Boso Bosch & Sohn GmbH, Jungingen, Germany) in order to calculate the pulse pressure. Maximal force of the untrained hand was measured with a digital hand dynamometer (SAEHAN Corp., Changwon city, Korea). All measurements were performed as triplicate. Venous blood samples were taken using K_3_-EDTA and Li-heparin S-Monovettes® (Sarstedt, Nümbrecht, Germany). Plasma was separated from the blood by centrifugation at 2000×***g*** for 10 min and then stored at −150°C. Lysozyme levels were measured in the K_3_-EDTA plasma with an ELISA (K6902; Immundiagnostik, Bensheim, Germany).

### NAD detection

NAD levels were analyzed by use of the colorimetric total NAD/NADH assay (ab186032; Abcam, Cambridge, U.K.) according to another protocol (see ab65348; Abcam) that allows the differentiation of NAD^+^ and NADH. The assay reagent consisted of a mixture of NAD^+^-reducing and NADH-oxidizing enzyme causing an amplification of the NAD signal. As NAD^+^ but not NADH is heat labile at already 60°C, total NAD is detected in the unheated sample and NADH is detected in the heated sample. The difference between total NAD and NADH represented the NAD^+^ amount. The different heat lability of NAD^+^ and NADH was proved by high-performance liquid chromatography (HPLC) analysis (see below).

In detail, plasma samples were loaded on 10 kDa Pierce™ protein concentrators (Thermo Fisher Scientific, Waltham, MA) and centrifuged at 13000×***g*** for 30 min (4°C) to remove proteins. As mentioned by the manufacturer and also tested by us, this separation step is essential for the NAD detection. One part of the filtrate was heated at 60°C for 30 min, whereas another part was kept on ice. A total of 20 µl of the unheated or heated filtrates as well as NADH (0.1–5.0 µM dissolved in PBS) were transferred to a clear 348-well microplate. After adding 20 µl of the enzyme reaction mix the color of produced formazan was measured at 460 nm in the infinite M1000 plate reader (Tecan, Männedorf, Switzerland) for 120 min at 1-min intervals. All samples were measured at least fourfold. In contrast with the assay protocol suggesting an end-point measurement, we calculated the increase in the signal intensity per time for the steady state period of the enzymatic cycling reaction.

**Figure 1:**
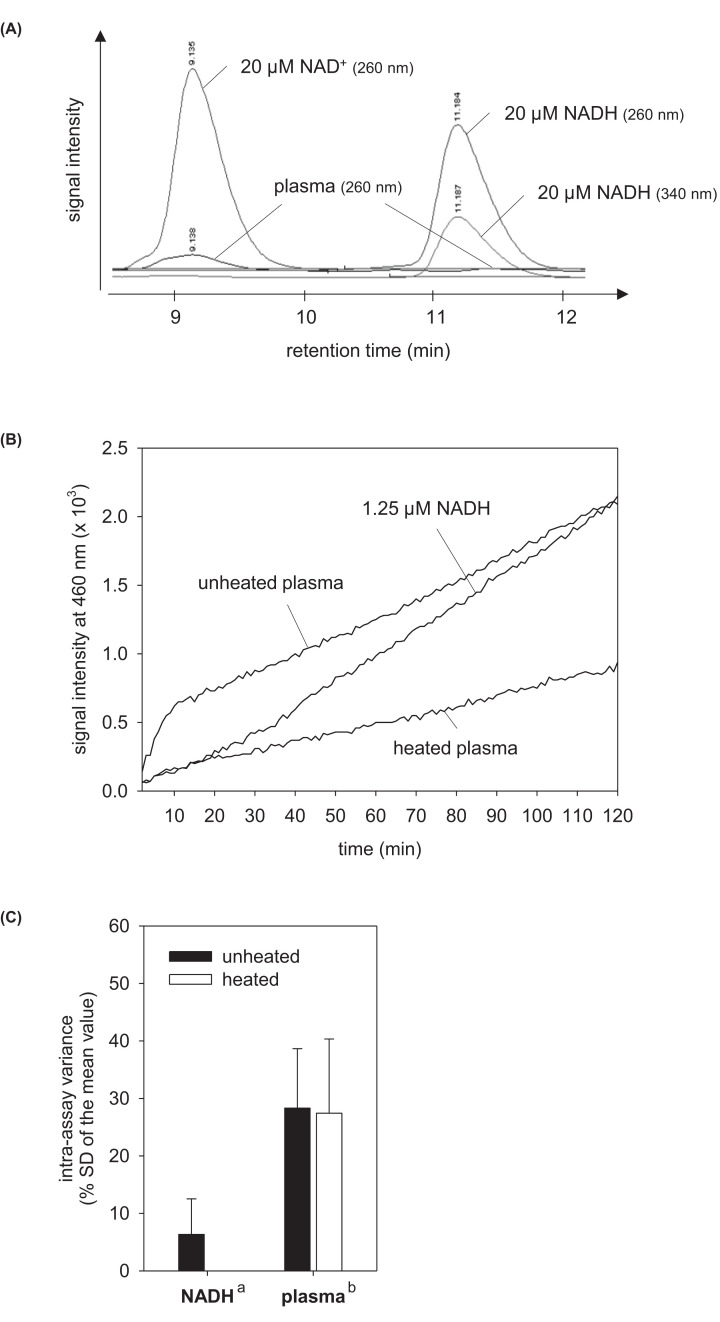
Establishment of NAD detection in human plasma HPLC chromatograms of filtered human plasma alone and spiked with 20 µM NAD^+^ and NADH (**A**). Enzymatic amplification reaction for pure NADH, unheated and heated plasma samples (**B**). Intra-assay variances of the NAD enzymatic amplification reaction for NADH, unheated and heated plasma measured fourfold (**C**). Chromatograms taken at 260 and 340 nm are shown as overlay. Data are shown as mean ± SD. ^a^ calculated from the NADH standard at 1.25 µM for all assay plates performed (n = 15), ^b^ calculated from all samples analyzed (n = 205).

In addition, NAD levels of some filtered plasma samples were analyzed in a 1200 series HPLC system equipped Zorba Eclipse xD13-C18 column and chemStation software (all compounds from Agilent Technologies, Santa Clara, CA) according to the protocol of Sporty et al. (2008) [39]. Pure NADH (N4505; Sigma–Aldrich, St. Louis, MO) and NAD^+^ (N1636; Sigma–Aldrich) were used as references. NADH can be detected at 260 and 340 nm, whereas NAD^+^ is detectable at 260 nm only (Figure 1A).

### Statistics

Statistical calculations were performed by use of the SigmaStat 3.5 software (Systat Software Inc., San Jose, CA). Depending on the results of the Normality distribution test, we used the Student’s *t* test or the Rank sum-test for comparing two groups. Age dependencies were tested by quadratic polynomial regression analysis because linear regression model analysis did not fit for these data. *P*-values ≤0.05 indicate significant changes.

## Results

### Study population

Our study included 91 men and 114 women. They were nearly equally distributed according to their chronological age and showed expected sex-dependent differences (i.e. hand force, medical drugs but not more antihypertensives; Table 1). Regression analyses revealed only few differences in the age dependency of selected parameters between men and women (Table 1). Among the two parameters representing the biological age, the skin autofluorescence correlated best with chronological age of men while the brachial-femoral PWV correlated best with chronological age of women (Table 1). Other clinical parameters collected were unsuited as biomarker of age (Table 1). None of the probands had bacterial infections as indicated by the low plasma lysozyme concentrations (Table 1).

### NAD detection assay

Initial analysis by HPLC identified a detection threshold of approximately 1–2 µM for pure NAD^+^ and NADH by this technology. As the NAD concentrations in plasma samples tested were around, often below, this threshold (Figure 1A) we established an enzymatic amplification assay being more sensitive than HPLC. In this assay, plasma samples showed a hyperbolic increase in the signal intensity in the first phase of the enzymatic amplification reaction followed by a linear increase (steady state) in the second phase (Figure 1B). This curve progression has not been seen for pure NADH (Figure 1B), NAD^+^ or a mixture of both (data not shown). The data evaluation within the hyperbolic phase of the enzymatic reaction yielded unrealistically high plasma NAD concentrations (>10 µM which must had been detected by HPLC), whereas the evaluation in the steady state phase did not. Therefore, all NAD concentrations were calculated according to the steady state phase of the enzymatic assay. The intra-assay variance of our biological samples has been determined with approximately 30% of the mean value, which is higher than that of pure NADH (Figure 1C).

### NAD concentrations in human plasma

**Figure 2:**
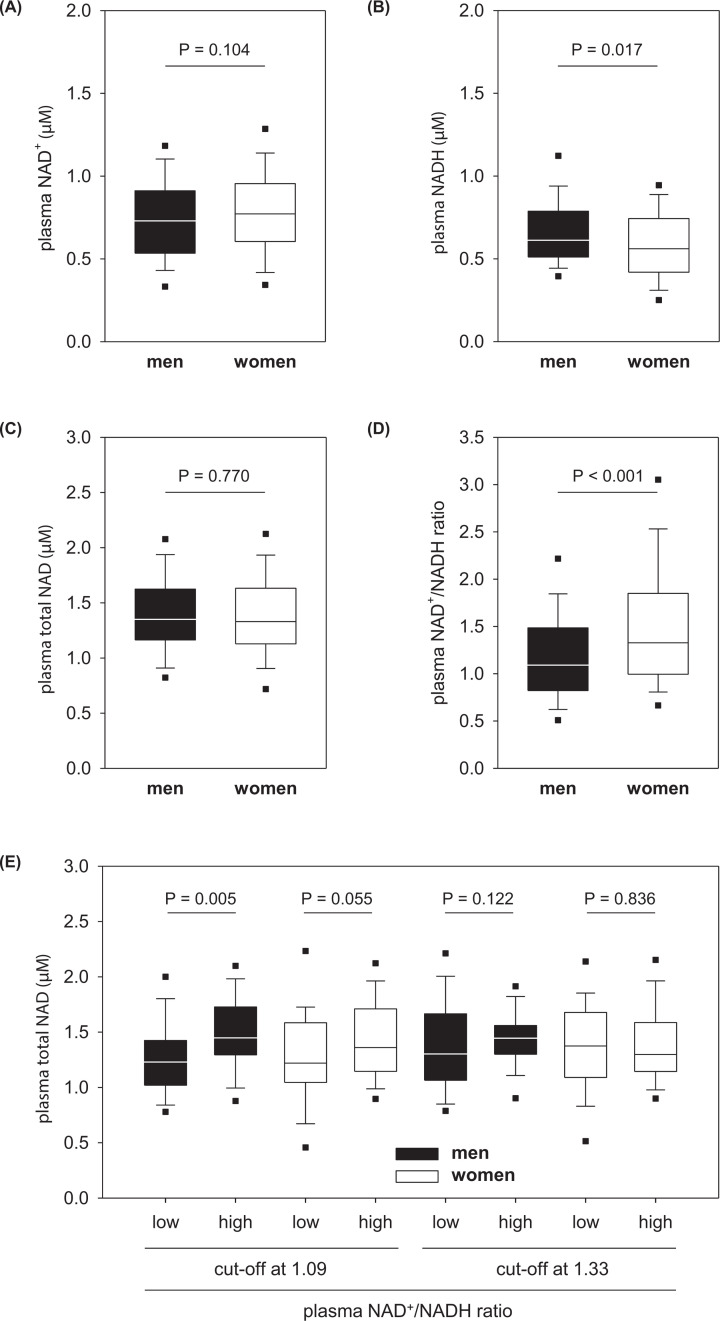
Sex dependency of the plasma NAD level Blood plasma concentration of NAD^+^ (**A**) NADH, (**B**) and total NAD (**C**), as well as the plasma NAD redox ratio (**D**) in men and women of a normal human population. Plasma total NAD depending on the median NAD^+^/NADH ratio for men (1.09) and women (1.33) (**E**). All data are shown as box plot with whiskers and 5^th^/95^th^ percentiles. In E, the subgroups contained n = 45, 46, 64 and 27 men as well as 37, 77, 57 and 57 women (black or with plots from left to right). P values were calculated by the Rank sum test.

In human plasma, the amounts of NAD^+^ and NADH are nearly balanced (Figure 2A,B). The total plasma NAD concentration was determined with median 1.34 µM (0.44–2.88 µM) for all probands without difference between men and women (Figure 2C). In contrast, the redox ratio of plasma NAD^+^/NADH was significantly higher in women than men (Figure 2D) due to their slightly higher values for NAD^+^ and lower values for NADH (Figure 2A,B). Therefore, we divided all men and women according to the sex-specific median values for the plasma NAD^+^/NADH ratio in subgroups followed by the statistical evaluation of all parameters collected. This subgroup analysis identified lower total NAD concentrations in the plasma samples of the low NAD^+^/NADH ratio subgroup, an effect that was much more pronounced in men than women (Figure 2E). However, men and women having lower plasma NAD^+^/NADH ratios did not show any other distinctive features.

**Figure 3:**
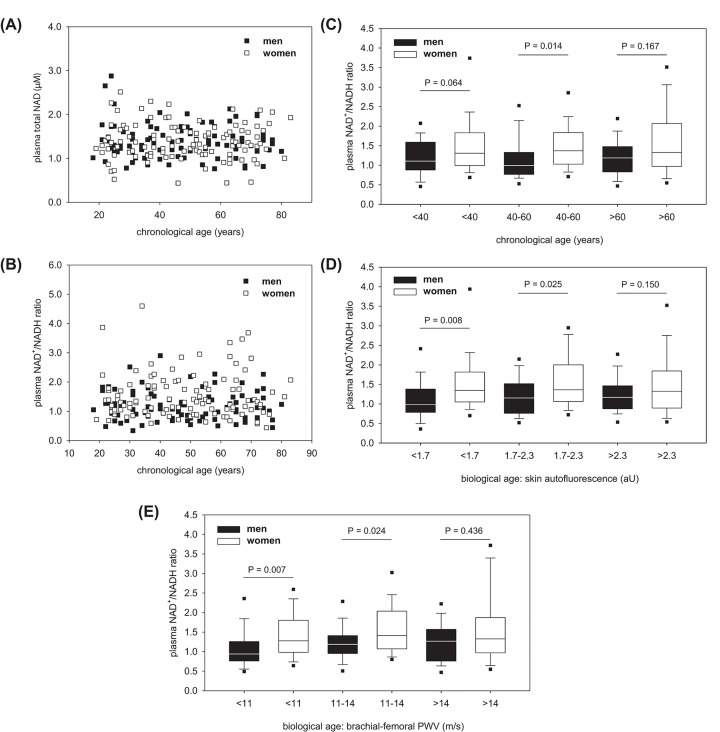
Age dependency of the plasma NAD level Plasma values for total NAD (**A**) and the NAD redox ratio (**B**) depending on chronological age of men and women. Data are shown as dot plots. No regression found. Influence of chronological (**C**) and biological age (**D,E**) on the sex-related plasma NAD^+^/NADH ratios. All data are shown as box plot with whiskers and 5th/95th percentiles. In (C), the subgroups contained *n*=36, 31 and 24 men as well as 41, 38 and 35 women (black or with plots from left to right). In (D), the subgroups contained *n*=22, 43 and 26 men as well as 38, 42 and 34 women. In (E), the subgroups contained *n*=31, 38 and 22 men as well as 42, 37 and 35 women. *P*-values were calculated by the Rank-sum test.

Age-dependent analyses did not show any correlation between chronological age of men or women and the plasma values for total NAD (Figure 3A) or NAD^+^/NADH ratio (Figure 3B). Skin autofluorescence or brachial-femoral PWV did also not correlate with the plasma values for total NAD or NAD^+^/NADH ratio (data not shown). However, Figure 3B suggests that the increased NAD^+^/NADH ratio in women (Figure 2D) mainly relates to middle-aged probands. This suggestion has been confirmed by subgrouping our study population in three ranges of the chronological age (Figure 3C). When subgrouping our study population according to our two markers of the biological age, the sex-related NAD^+^/NADH ratio decreases with higher values for the skin autofluorescence (Figure 3D) and brachial-femoral PWV (Figure 3E). Women older than 60 years or having skin autofluorescence values above 2.3 or brachial-femoral PWVs faster than 14 m/s do not show higher NAD^+^/NADH ratio than men anymore (Figure 3D,E).

## Discussion

The present study showed that human plasma contains low micromolar concentrations of total NAD. Neither sex nor increasing age influenced the total amount of plasma NAD in a normal human population having no severe diseases. The plasma concentration of its oxidized form, NAD^+^, was somewhat higher than that of its reduced form, NADH. Especially young and middle-aged women had more plasma NAD^+^ than NADH, whereas the sex-related difference in the plasma NAD^+^/NADH ratio was abolished at older ages.

The number of scientific articles investigating extracellular NAD in human is still very low. Analysis of extracellular NAD in other species are lacking at all. In total, only five publications [5,16,17,36] and one congress report [37] have been identified in the literature database. Moreover, the blood levels of extracellular NAD described vary extremely (0.05–500 µM) [5,16,17,36]. We identified plasma concentrations between 0.44 and 2.88 µM which corresponds to the values described by Singhal and Zhan (2006) in a congress report (0.1–1.2 µM) [37]. Consistent with our analysis, they also investigated human blood samples by an enzymatic assay and confirmed the range measured by HPLC technology [37]. Another study described much less NAD in human plasma without mentioning the assay procedure [36]. In contrast, a clinical research group identified much higher plasma or serum values for NAD by enzymatic analysis [5,16,17]. Reason for these high concentrations of NAD calculated might be their assay protocol because they did not remove proteins from the plasma by filtration, which is an essential step in the plasma NAD detection. Also, they did an end-point measurement after already 10 min. At this time, the NAD enzymatic amplification reaction of plasma samples is still within the hyperbolic phase of the reaction (see Figure 1B). It seems that low molecular weight factors remaining in the filtered plasma accelerate the NAD enzymatic amplification reaction at the beginning, an effect that is abolished after their consumption sometimes later. This specific case shows that measurements of reaction curves are more precise than end-point measurements when using enzymatic assay tests. Nevertheless, enzymatic assays will always have the disadvantage that unknown factors can influence the enzyme activity. The presence of assay-influencing factors in the plasma is also suggested by the higher intra-assay variance for plasma samples than pure NADH. However, a more direct measurement of the plasma NAD status by HPLC technology had not been better because of its low sensitivity.

Cx43 hemichannels release intracellular NAD^+^ into the extracellular space [6]. Therefore, plasma NAD^+^ might result from the Cx43 activity of Cx43-expressing cells, especially cardiomyocytes and nerve cells [27]. As NADH can also be detected in human plasma, it suggests the existence of extracellular NAD^+^ reductases as either soluble enzymes or ectoenzymes. However, their identity has not yet been described so far which is in contrast with extracellular enzymes having NADH-oxidizing activity. Although the contribution of extracellular NADH oxidases to the reversal of plasma NADH to NAD^+^ is still unknown, such oxidases could be the ecto-NOX disulfide-thiol exchangers 1 and 2 [9,20] or renalase [29]. Of course, occasional lysis of cells could also contribute to the plasma amounts of NAD^+^ as well as NADH.

The relative amount of plasma NAD^+^ per NADH is higher in women than men. This sex-related difference has also been described in healthy human individuals (46–78 years old) by Singhal and Zhan (2006) in their congress report [37]. As mechanisms regulating the NAD^+^/NADH redox ratio outside the cells are largely unknown, the biological background of the sex-related difference in the plasma NAD^+^/NADH ratio is hardly discussable. One potential reason indirectly affecting the plasma NAD^+^/NADH ratio could be a lower NAD^+^ release through Cx43 hemichannels and/or higher metabolization of NAD^+^ through NAD^+^-consuming enzymes like CD38 in men. This assumption is supported by our observation that especially men having low plasma NAD^+^/NADH ratios have also lower plasma concentrations of total NAD. Moreover, NAD^+^/NADH-regulating mechanisms must depend on age as the sex-related difference in plasma NAD^+^/NADH were abolished in old probands. It is well conceivable that female sex hormones and differences between pre- and postmenopausal phase in women contribute to the general difference in the plasma NAD^+^/NADH ratio between men and women and its loss with higher age. However, experimental evidences are still missing. In addition to hormonal differences, also sex- and age-related differences in the dietary intake of NAD^+^ precursors eventually contribute to the different plasma NAD^+^/NADH ratios observed.

Initially, we assumed a significant effect of age on the plasma levels of total NAD and NAD^+^/NADH ratio. One reason for this assumption had been the lower activity of the salvage pathway with increasing age and, therefore, lower resynthesis of NAD^+^ from NAD metabolites [19]. Another reason had been the effect of age on the tissue level of CD38 and Cx43 [8,11,32]. However, aging did not alter the plasma levels of total NAD and NAD^+^/NADH ratio in humans. This observation suggests that mechanisms behind the plasma amount of NAD are very complex. Although the Cx43 down-regulation and CD38 up-regulation in tissues might cause lower plasma NAD amounts, this effect could be abolished in old organisms in case of more cell lysis. Nevertheless, the age-related decline in the difference of the plasma NAD^+^/NADH ratio between men and women suggests a certain effect of increasing age on the regulation of extracellular NAD. This suggestion is also supported by the fact that selected markers of the biological age indicated the sex-related difference of the plasma NAD^+^/NADH ratio and its decline with higher biological age somewhat better than with higher chronological age. In our specific case, the usefulness of biomarkers of aging became obvious for probands being in the early and middle adulthood. Nevertheless, our sub-evaluations suffer from a relatively low number of men and women each age group due to the total number of only 205 probands. Although multivariant statistical analyses had been possible to describe further age-related differences in the plasma NAD level between men and women, their real existence would be uncertain without additional confirmation by another independent study group.

Our study aimed at the analysis of the plasma NAD status in humans having, except for common age-related disorders, no severe acute or chronic diseases to detect relevant basal levels for total NAD and NAD^+^/NADH ratio in blood plasma. This is absolutely necessary in a research field that is not yet well investigated. However, only future studies will show if this plasma NAD status is influenced by acute or chronic diseases. Despite some technical limitations, others already indicated the influence of multiple sclerosis [5], dizziness [21] and congenital malformation [36] on the extracellular NAD^+^/NADH ratio in the extracellular fluid.

In summary, our study shows the normal levels of plasma NAD for the total NAD concentration and NAD^+^/NADH redox ratio depending on sex and age. Differences in the plasma NAD^+^/NADH ratio between men and women suggest sex-dependent variances in mechanisms regulating the extracellular NAD^+^ amount which still need to be identified.

## Data Availability

NAD datasets generated during the study are available from the corresponding author on reasonable request.

## Abbreviations

CD: cluster of differentiation
Cx43: connexin-43
HPLC: high-performance liquid chromatography
NAD: nicotinamide adenine dinucleotide
PWV: pulse wave velocity

## References

[1] Abdul-Salam V.B., Ramrakha P., Krishnan U., Owen D.R., Shalhoub J., Davies A.H. et al. (2010) Identification and assessment of plasma lysozyme as a putative biomarker of atherosclerosis. Arterioscler. Thromb. Vasc. Biol. 30, 1027–1033 10.1161/ATVBAHA.109.199810 20167661

[2] Alefishat E., Alexander S.P. and Ralevic V. (2015) Effects of NAD at purine receptors in isolated blood vessels. Purinergic Signal. 11, 47–57 10.1007/s11302-014-9428-1 25315718 PMC4336311

[3] Baier D., Teren A., Wirkner K., Loeffler M. and Scholz M. (2018) Parameters of pulse wave velocity: determinants and reference values assessed in the population-based study LIFE-Adult. Clin. Res. Cardiol. 107, 1050–1061 10.1007/s00392-018-1278-3 29766282 PMC6208658

[4] Belenky P., Bogan K.L. and Brenner C. (2007) NAD^+^ metabolism in health and disease. Trends Biochem. Sci. 32, 12–19 10.1016/j.tibs.2006.11.006 17161604

[5] Braidy N., Lim C.K., Grant R., Brew B.J. and Guillemin G.J. (2013) Serum nicotinamide adenine dinucleotide levels through disease course in multiple sclerosis. Brain Res. 1537, 267–272 10.1016/j.brainres.2013.08.025 23973746

[6] Bruzzone S., Guida L., Zocchi E., Franco L. and De Flora A. (2001) Connexin 43 hemichannels mediate Ca^2+^-regulated transmembrane NAD^+^ fluxes in intact cells. FASEB J. 15, 10–12 10.1096/fj.00-0566fje 11099492

[7] Burnstock G. (2017) Purinergic signaling in the cardiovascular system. Circ. Res. 120, 207–228 10.1161/CIRCRESAHA.116.309726 28057794

[8] Camacho-Pereira J., Tarrago M.G., Chini C.C.S., Nin V., Escande C., Warner G.M. et al. (2016) CD38 dictates age-related NAD decline and mitochondrial dysfunction through an SIRT3-dependent mechanism. Cell Metab. 23, 1127–1139 10.1016/j.cmet.2016.05.006 27304511 PMC4911708

[9] Chueh P.J., Kim C., Cho N., Morre D.M. and Morre D.J. (2002) Molecular cloning and characterization of a tumor-associated, growth-related, and time-keeping hydroquinone (NADH) oxidase (tNOX) of the HeLa cell surface. Biochemistry 41, 3732–3741 10.1021/bi012041t 11888291

[10] Connell N.J., Houtkooper R.H. and Schrauwen P. (2019) NAD^+^ metabolism as a target for metabolic health: have we found the silver bullet? Diabetologia 62, 888–899 10.1007/s00125-019-4831-3 30772929 PMC6509089

[11] Cotrina M.L., Gao Q., Lin J.H. and Nedergaard M. (2001) Expression and function of astrocytic gap junctions in aging. Brain Res. 901, 55–61 10.1016/S0006-8993(01)02258-2 11368950

[12] Da Moura Semedo C., Webb M., Waller H., Khunti K. and Davies M. (2017) Skin autofluorescence, a non-invasive marker of advanced glycation end products: clinical relevance and limitations. Postgrad. Med. J. 93, 289–294 10.1136/postgradmedj-2016-134579 28143896

[13] Garten A., Petzold S., Korner A., Imai S. and Kiess W. (2009) Nampt: linking NAD biology, metabolism and cancer. Trends Endocrinol. Metab. 20, 130–138 10.1016/j.tem.2008.10.004 19109034 PMC2738422

[14] Grabowska W., Sikora E. and Bielak-Zmijewska A. (2017) Sirtuins, a promising target in slowing down the ageing process. Biogerontology 18, 447–476 10.1007/s10522-017-9685-9 28258519 PMC5514220

[15] Grahnert A., Klein C. and Hauschildt S. (2009) Involvement of P2X receptors in the NAD^+^-induced rise in [Ca^2+^]_i_ in human monocytes. Purinergic Signal. 5, 309–319 10.1007/s11302-009-9144-4 19221895 PMC2717312

[16] Guest J., Grant R., Garg M., Mori T.A., Croft K.D. and Bilgin A. (2014) Cerebrospinal fluid levels of inflammation, oxidative stress and NAD^+^ are linked to differences in plasma carotenoid concentrations. J. Neuroinflammation 11, 117 10.1186/1742-2094-11-117 24985027 PMC4096526

[17] Guest J., Grant R., Mori T.A. and Croft K.D. (2014) Changes in oxidative damage, inflammation and [NAD(H)] with age in cerebrospinal fluid. PLoS ONE 9, e85335 10.1371/journal.pone.0085335 24454842 PMC3891813

[18] Imai S. and Guarente L. (2014) NAD^+^ and sirtuins in aging and disease. Trends Cell Biol. 24, 464–471 10.1016/j.tcb.2014.04.002 24786309 PMC4112140

[19] Imai S.I. and Guarente L. (2016) It takes two to tango: NAD^+^ and sirtuins in aging/longevity control. NPJ Aging Mech. Dis. 2, 16017 10.1038/npjamd.2016.17 28721271 PMC5514996

[20] Jiang Z., Morre D.M. and Morre D.J. (2006) A role for copper in biological time-keeping. J. Inorg. Biochem. 100, 2140–2149 10.1016/j.jinorgbio.2006.08.007 17027975

[21] Kao C.L., Tsai K.L., Cheng Y.Y., Kuo C.H., Lee S.D. and Chan R.C. (2014) Vestibular rehabilitation ameliorates chronic dizziness through the SIRT1 axis. Front. Aging Neurosci. 6, 27 10.3389/fnagi.2014.00027 24624081 PMC3941041

[22] Kim S.Y., Cohen B.M., Chen X., Lukas S.E., Shinn A.K., Yuksel A.C. et al. (2017) Redox dysregulation in Schizophrenia revealed by in vivo NAD^+^/NADH measurement. Schizophr. Bull. 43, 197–204 10.1093/schbul/sbw129 27665001 PMC5216857

[23] Klein C., Grahnert A., Abdelrahman A., Muller C.E. and Hauschildt S. (2009) Extracellular NAD^+^ induces a rise in [Ca^2+^]_i_ in activated human monocytes via engagement of P2Y_1_ and P2Y_11_ receptors. Cell Calcium. 46, 263–272 10.1016/j.ceca.2009.08.004 19748117

[24] Koch-Nolte F., Adriouch S., Bannas P., Krebs C., Scheuplein F., Seman M. et al. (2006) ADP-ribosylation of membrane proteins: unveiling the secrets of a crucial regulatory mechanism in mammalian cells. Ann. Med. 38, 188–199 10.1080/07853890600655499 16720433

[25] Lee S.Y. and Muller C.E. (2017) Nucleotide pyrophosphatase/phosphodiesterase 1 (NPP1) and its inhibitors. Medchemcomm 8, 823–840 10.1039/C7MD00015D 30108800 PMC6072468

[26] Malavasi F., Deaglio S., Funaro A., Ferrero E., Horenstein A.L., Ortolan E. et al. (2008) Evolution and function of the ADP ribosyl cyclase/CD38 gene family in physiology and pathology. Physiol. Rev. 88, 841–886 10.1152/physrev.00035.2007 18626062

[27] Marquez-Rosado L., Solan J.L., Dunn C.A., Norris R.P. and Lampe P.D. (2012) Connexin43 phosphorylation in brain, cardiac, endothelial and epithelial tissues. Biochim. Biophys. Acta 1818, 1985–1992 10.1016/j.bbamem.2011.07.028 21819962 PMC3241956

[28] Massudi H., Grant R., Braidy N., Guest J., Farnsworth B. and Guillemin G.J. (2012) Age-associated changes in oxidative stress and NAD^+^ metabolism in human tissue. PLoS ONE 7, e42357 10.1371/journal.pone.0042357 22848760 PMC3407129

[29] Moran G.R. (2016) The catalytic function of renalase: a decade of phantoms. Biochim. Biophys. Acta 1864, 177–186 10.1016/j.bbapap.2015.04.010 25900362

[30] Moreschi I., Bruzzone S., Nicholas R.A., Fruscione F., Sturla L., Benvenuto F. et al. (2006) Extracellular NAD^+^ is an agonist of the human P2Y11 purinergic receptor in human granulocytes. J. Biol. Chem. 281, 31419–31429 10.1074/jbc.M606625200 16926152

[31] Mutafova-Yambolieva V.N., Hwang S.J., Hao X., Chen H., Zhu M.X., Wood J.D. et al. (2007) Beta-nicotinamide adenine dinucleotide is an inhibitory neurotransmitter in visceral smooth muscle. Proc. Natl. Acad. Sci. U.S.A. 104, 16359–16364 10.1073/pnas.0705510104 17913880 PMC2042211

[32] Nagibin V., Egan Benova T., Viczenczova C., Szeiffova Bacova B., Dovinova I., Barancik M. et al. (2016) Ageing related down-regulation of myocardial connexin-43 and up-regulation of MMP-2 may predict propensity to atrial fibrillation in experimental animals. Physiol. Res. 65, S91–S100 10.33549/physiolres.933389 27643943

[33] Okabe K., Yaku K., Tobe K. and Nakagawa T. (2019) Implications of altered NAD metabolism in metabolic disorders. J. Biomed. Sci. 26, 34 10.1186/s12929-019-0527-8 31078136 PMC6511662

[34] Rossi L., Salvestrini V., Ferrari D., Di Virgilio F. and Lemoli R.M. (2012) The sixth sense: hematopoietic stem cells detect danger through purinergic signaling. Blood 120, 2365–2375 10.1182/blood-2012-04-422378 22786880

[35] Roth G.A., Huffman M.D., Moran A.E., Feigin V., Mensah G.A., Naghavi M. et al. (2015) Global and regional patterns in cardiovascular mortality from 1990 to 2013. Circulation 132, 1667–1678 10.1161/CIRCULATIONAHA.114.008720 26503749

[36] Shi H., Enriquez A., Rapadas M., Martin E., Wang R., Moreau J. et al. (2017) NAD deficiency, congenital malformations, and niacin supplementation. N. Engl. J. Med. 377, 544–552 10.1056/NEJMoa1616361 28792876

[37] Singhal R.P. and Zhan J.J. (2006) NAD^+^ and NADH concentrations in younger and older human adults. FASEB J. 20, A1357–A1357

[38] Song E.K., Rah S.Y., Lee Y.R., Yoo C.H., Kim Y.R., Yeom J.H. et al. (2011) Connexin-43 hemichannels mediate cyclic ADP-ribose generation and its Ca^2+^-mobilizing activity by NAD^+^/cyclic ADP-ribose transport. J. Biol. Chem. 286, 44480–44490 10.1074/jbc.M111.307645 22033928 PMC3247979

[39] Sporty J.L., Kabir M.M., Turteltaub K.W., Ognibene T., Lin S.J. and Bench G. (2008) Single sample extraction protocol for the quantification of NAD and NADH redox states in Saccharomyces cerevisiae. J. Sep. Sci. 31, 3202–3211 10.1002/jssc.200800238 18763242 PMC2640230

[40] Stein L.R. and Imai S. (2012) The dynamic regulation of NAD metabolism in mitochondria. Trends Endocrinol. Metab. 23, 420–428 10.1016/j.tem.2012.06.005 22819213 PMC3683958

[41] Zhou C.C., Yang X., Hua X., Liu J., Fan M.B., Li G.Q. et al. (2016) Hepatic NAD^+^ deficiency as a therapeutic target for non-alcoholic fatty liver disease in ageing. Br. J. Pharmacol. 173, 2352–2368 10.1111/bph.13513 27174364 PMC4945761

[42] Zhu X.H., Lu M., Lee B.Y., Ugurbil K. and Chen W. (2015) In vivo NAD assay reveals the intracellular NAD contents and redox state in. healthy human brain and their age dependences. Proc. Natl. Acad. Sci. U.S.A. 112, 2876–2881 10.1073/pnas.1417921112 25730862 PMC4352772

